# Susceptibility of *Mycobacterium tuberculosis*-infected host cells to phospho-MLKL driven necroptosis is dependent on cell type and presence of TNFα

**DOI:** 10.1080/21505594.2017.1377881

**Published:** 2017-11-24

**Authors:** Rachel E. Butler, Nitya Krishnan, Waldo Garcia-Jimenez, Robert Francis, Abbe Martyn, Tom Mendum, Shaza Felemban, Nicolas Locker, Francisco J. Salguero, Brian Robertson, Graham R. Stewart

**Affiliations:** a Department of Microbial and Cellular Sciences, Faculty of Health and Medical Sciences, University of Surrey, Guildford, Surrey, UK; b MRC Centre for Molecular Bacteriology and Infection, Department of Medicine, Flowers Building, Imperial College London, South Kensington, London, UK; c Department of Pathology and Infectious Diseases, School of Veterinary Medicine, University of Surrey, Guildford, Surrey, UK

**Keywords:** fibroblast, macrophage, MLKL, *Mycobacterium tuberculosis*, necroptosis, RIPK1, RIPK3

## Abstract

An important feature of *Mycobacterium tuberculosis* pathogenesis is the ability to control cell death in infected host cells, including inhibition of apoptosis and stimulation of necrosis. Recently an alternative form of programmed cell death, necroptosis, has been described where necrotic cell death is induced by apoptotic stimuli under conditions where apoptotic execution is inhibited. We show for the first time that *M. tuberculosis* and TNFα synergise to induce necroptosis in murine fibroblasts via RIPK1-dependent mechanisms and characterized by phosphorylation of Ser345 of the MLKL necroptosis death effector. However, in murine macrophages *M. tuberculosis* and TNFα induce non-necroptotic cell death that is RIPK1-dependent but independent of MLKL phosphorylation. Instead, *M. tuberculosis*-infected macrophages undergo RIPK3-dependent cell death which occurs both in the presence and absence of TNFα and involves the production of mitochondrial ROS. Immunocytochemical staining for MLKL phosphorylation further demonstrated the occurrence of necroptosis *in vivo* in murine *M. tuberculosis* granulomas. Phosphorylated-MLKL immunoreactivity was observed associated with the cytoplasm and nucleus of fusiform cells in *M. tuberculosis* lesions but not in proximal macrophages. Thus whereas pMLKL-driven necroptosis does not appear to be a feature of *M. tuberculosis*-infected macrophage cell death, it may contribute to TNFα-induced cytotoxicity of the lung stroma and therefore contribute to necrotic cavitation and bacterial dissemination.

## Introduction

*Mycobacterium tuberculosis* is an intracellular pathogen that resides predominantly in macrophages but also in other cells including dendritic cells and non-professional phagocytes such as fibroblasts, adipocytes and endothelial cells.^1,2,3^ The bacterium has evolved sophisticated and robust systems to control the biology of its host cell; preserving its replicative niche, avoiding innate antimicrobial mechanisms and manipulating the generation of adaptive immunity.^4,5^ The fine control of inflammation is particularly important for *M. tuberculosis* because the bacterium must avoid stimulation of immunity that will limit its infection whilst maintaining the immune driven generation of a necrotic pulmonary granuloma, cavitation and subsequent respiratory transmission.

An important component of *M. tuberculosis* pathogenesis is the complex control over the mode and timing of host cell death. In general terms, macrophages infected with *M. tuberculosis* may undergo cell death by two mechanisms, apoptosis or necrosis, with drastically different outcomes for the host and bacterium. Several studies have demonstrated that apoptosis of infected macrophages results in killing of mycobacteria,^6–10^ probably by efferocytosis of mycobacteria-containing apoptotic bodies and subsequent lysosomal digestion or oxidative killing.^11,12^ Additionally, macrophage apoptosis stimulates protective T cell responses through the “detour” pathway of antigen presentation.^13–15^ In contrast, necrosis has been observed to facilitate release of viable bacteria from infected macrophages^8,16^ which may be taken up by phagocytes attracted by damage associated molecular patterns (DAMPs) released by the necrotic macrophage.^17,18^ This would allow further intracellular replication producing a cycle of host cell infection, necrosis and reinfection that may represent an important part of the generation of necrotic granuloma. Indeed, stimulation of necrosis is a hallmark of virulent mycobacterial strains^16,19,20^ and as such stimulation of necrosis is considered a virulence mechanism of *M. tuberculosis*.

In more specific terms, it has become apparent that *M. tuberculosis* is able to exert an exquisitely complex control over cell death of the host cell, by having the capacity to both induce and inhibit apoptosis and induce necrosis of the host cell. Apoptosis can be induced by the extrinsic (death receptor) or intrinsic (mitochondrial) pathways. *M. tuberculosis* is able to inhibit tumour necrosis factor alpha (TNFα)-mediated extrinsic apoptosis via a number of mechanisms including secretion of soluble TNF receptor 2 (sTNFR2),^21^ downregulation of pro-caspase-8 transcription,^22^ suppression of caspase-8 expression,^23^ and upregulation of caspase-8-inhibiting FLIP molecules transcription.^22^ However, inhibition of the extrinsic pathway occurs in the context of activation of the intrinsic mitochondrial pathway.^23^ During infection with avirulent mycobacterial strains such as H37Ra, mitochondrial outer membrane permeablisation and release of cytochrome C lead to host cell apoptosis.^23^ However virulent mycobacterial strains such as H37Rv induce irreversible mitochondrial inner membrane permeablisation, leading to mitochondrial permeability transition (MPT), causing further loss of mitochondrial integrity and function.^23^ This, plus further mechanisms inhibiting plasma membrane repair,^24^ leads to necrosis of the macrophage. Thus a model of macrophage infection has emerged where mycobacteria preserve themselves and their macrophage hosts by inhibition of apoptosis and then exit the cell to disseminate further via necrosis.

Necrosis of cells can be induced by a variety of cellular stresses and until recently was considered to be a disordered mode of death that did not involve intracellular signalling pathways. However, in the last decade, highly coordinated modes of *programmed* necrotic cell death have been described. Necroptosis is a pharmacologically tractable necrosis,^25^ that can be induced by death receptors including TNFR1,^26,27^ type I interferon,^28^ and recognition of pathogen-associated molecular patterns (PAMPS) by pattern recognition receptors including toll-like receptors TLR3, TLR4, and the cytosolic DNA-dependent activator or IFN regulatory factors DAI/ZBP1.^29^ Necroptosis occurs when cell death is induced by apoptotic stimuli under conditions where apoptotic execution is inhibited. In the case of TNFα-stimulated necroptosis, when TNFα signalling occurs in the presence of caspase inhibition (such as the pan caspase inhibitor zVAD.fmk^30^), the receptor interacting kinases RIPK1 and RIPK3 associate and become phosphorylated and the pseudokinase mixed lineage kinase domain-like protein (MLKL) is recruited and phosphorylated by pRIPK3.^27,31,32^ The resulting complex translocates to the nucleus and then to the cell membrane where oligomerized pMLKL has pore forming activity and causes necrotic cell lysis.^33^ Necroptosis can be inhibited using the RIPK1 inhibitor necrostatin-1 (Nec-1).^34,35^ RIPK1 also plays a role in cell survival by limiting capsase-8 and TNFR-induced apoptosis,^36^ as demonstrated by perinatal lethality in *ripk1*^−/−^ mice.^37^ Additionally, RIPK1 in complex with RIPK3, FADD and caspase-8 can mediate apoptosis; as such RIPK1 dependence of cell death (such as cell death that can be inhibited by Nec-1) does not in itself confirm necroptosis as a mechanism.^25^

Necroptosis has been described in a number of pathological conditions with overt inflammatory signatures including Crohn's disease,^38^ and acts as a defence mechanism against some viral pathogens such as Vaccinia virus and murine cytomegalovirus.^26,39,40^ Accordingly, viruses have evolved mechanisms to inhibit necroptosis to counter this mechanism.^40,41^ Necroptosis has also been observed in bacterial infections including Salmonella and Listeria, where its induction was associated with loss of immune control and increased pathogen replication.^28,42^ More recently, programmed necrosis was reported in *Mycobacterium marinum* infection of leukotriene A4 hydrolase (LTA4H) mutant zebrafish, which express high levels of TNFα.^43^

TNFα is a pivotal cytokine in tuberculosis, being essential for protection but, paradoxically, at high levels also responsible for the generation of tissue necrosis, increased tissue pathology and enhanced bacterial growth.^43–46^ Given that *M. tuberculosis* is able to inhibit extrinsic apoptosis pathways in the context of high circulating levels of TNFα, our focus was drawn to the potential role of TNFα stimulated necroptosis in *M. tuberculosis* infection.

Much of what we know about *M. tuberculosis* host cell death has been gained from studies of macrophage infection. However, it has long been established that infection with *M. tuberculosis* sensitises fibroblasts to TNFα toxicity.^47,48^ Of further intrigue, infected fibroblasts are seen in the lungs of cadavers with latent *M. tuberculosis* infection but are not seen during active *M. tuberculosis* infection.^2^ An enhanced sensitivity of infected fibroblasts to TNFα has been postulated to account for this but no mechanistic detail of the mode of cell death has been elucidated.^48^

Given the prominent role of TNFα in tuberculosis and the capacity of *M. tuberculosis* to inhibit apoptosis in macrophages and sensitize fibroblasts the toxic effects of TNFα, we hypothesised that necroptosis may occur during *M. tuberculosis* infection and may represent an important mode of necrotic cell death. Thus, we investigated the occurrence of necroptosis in infected murine macrophage and fibroblast cells in the presence of TNFα. To definitively demonstrate the presence or absence of necroptosis, we determined the occurrence of phosphorylated MLKL in cells exposed to TNFα, and *in vivo* in murine granulomatous *M. tuberculosis* lesions.

## Results

**Figure 1.**
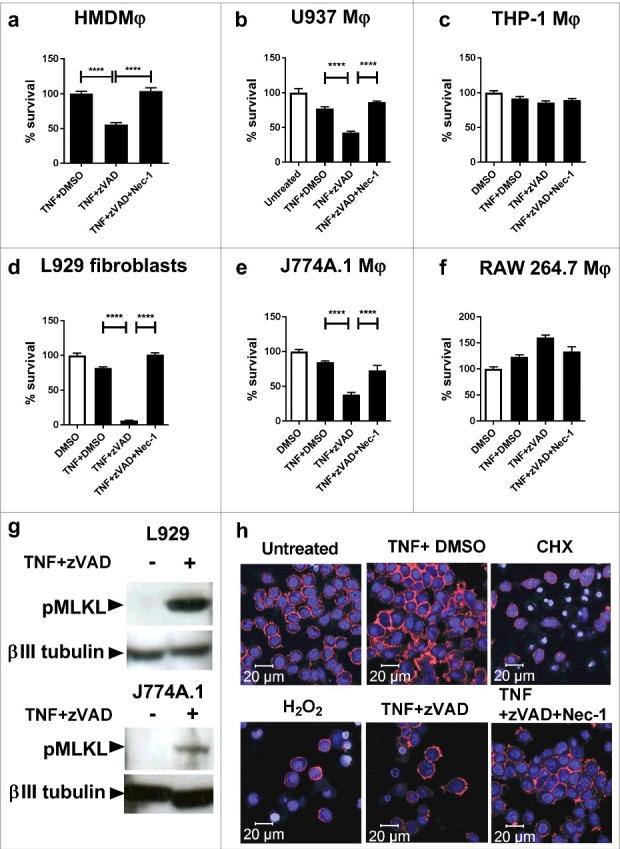
Necroptosis of macrophage cell lines, human MDMs and murine fibroblasts treated with TNFα+zVAD. (a) Human monocyte-derived macrophages (HMDMφ) were treated for 20 hours with 50ng/mL TNF+DMSO, TNF+30 µM zVAD and TNF+30 µM zVAD + 30 µM Nec-1, before measuring cell survival using a crystal violet assay (normalised to TNFα+DMSO treated control cells). Results are mean +/- SEM n = 4, and are representative of 3 independent experiments. (b) U937 macrophages were treated for 20 hours with 50ng/mL TNF, TNF+30 µM zVAD and TNF+30 µM zVAD + 30 µM Nec-1, before measuring cell survival using a crystal violet assay (normalised to untreated control cells). Results are mean +/- SEM n = 10, and are representative of at least 2 independent experiments. (c) THP-1 were treated for 20 hours with 50ng/mL TNF+DMSO, TNF+30 µM zVAD, and TNF + 30 µM zVAD + 30 µM Nec-1, before measuring cell survival using a crystal violet assay (normalised to DMSO treated control cells). Results are mean +/- SEM n = 10. (d) L929 fibroblasts, (e) J774A.1 macrophages and (f) RAW macrophages were treated for 20 hours with 10ng/ml TNF+ DMSO, TNF + 30 µM zVAD, and TNF + 30 µM zVAD + 30 µM Nec-1. Results are mean +/- SEM n = 10, are expressed as a percentage of DMSO treated controls, and representative of 2–3 independent experiments. Statistics are one way ANOVA with Tukey's post-test. ****p<0.0001. (g) Western blot of lysates of L929 fibroblasts and J774A.1 macrophages that had been treated with 10ng/ml TNFα and 25ng/ml TNFα respectively in the presence of 30 µM zVAD.fmk for 18 hours, developed with antibodies against MLKL phosphorylated at Ser345, or βIII tubulin as a loading control. Results are representative of 2–3 independent experiments. (h) J774A.1 macrophages were seeded on glass slide flasks and untreated or treated with cyclohexamide 2.5 µg/ml, 0.5mM H_2_O_2_, TNFα 25ng/ml, TNF + 30 µM zVAD, and TNF + 30 µM zVAD + 30 µM Nec-1. After 20 hours, cells were stained with Alexa-568-phalloidin (displayed as red) and counterstained with DRAQ5 (displayed as blue) before viewing by confocal microscopy. Results are representative of 2 independent experiments.

In order to investigate the presence of necroptosis in response to *M. tuberculosis* infection, we first compared the capability of murine fibroblasts and human and murine macrophages to undergo necroptosis induced by TNFα + zVAD treatment, as this capacity is not universal in eukaryotic cells.^27^ Monolayers of cells (plus control wells) were treated with TNFα (plus DMSO control), TNFα+zVAD, or TNFα+zVAD+Nec-1. After 20 hours, cell survival was determined by crystal violet assay. As seen in Fig. 1a-b, primary human monocyte-derived macrophages and U937 macrophages underwent cell death in response to TNFα+zVAD treatment, and this could be inhibited by the RIPK1 inhibitor Nec-1. However, THP-1 macrophages (Fig. 1c) were not sensitive to TNFα+zVAD treatment. In the murine system, L929 fibroblasts (a cell type well characterised in its ability to undergo necroptosis),^27^ and J774A.1 macrophages underwent cell death in response to TNFα+zVAD treatment, and cell death could be inhibited by Nec-1 (Fig. 1d-e). However, RAW 264.7 macrophages did not share the capacity to undergo cell death stimulated by TNFα+zVAD treatment (Fig. 1f).

We focussed on characterising cell death in murine L929 fibroblasts and murine J774A.1 macrophages. As seen in Fig. 1g, cell death induced by TNFα+zVAD treatment in both L929 fibroblasts and J774A.1 macrophages was confirmed to be necroptosis by detection by Western blot of phosphorylation of MLKL at Ser345, which represents an essential step in the canonical effector mechanism of necroptotic death.^49^ We further sought to characterise the cell death characteristics of J774A.1 macrophages undergoing necroptosis. J774A.1 cells were induced to undergo apoptosis (by treatment with cyclohexamide), necrosis (by H_2_O_2_-treatment) and necroptosis (by TNFα+zVAD treatment) and examined by confocal microscopy. As seen in Fig. 1h, cells undergoing necroptosis undergo necrotic cell death that lacks the apoptotic hallmarks of nuclear condensation and fragmentation.

**Figure 2.**
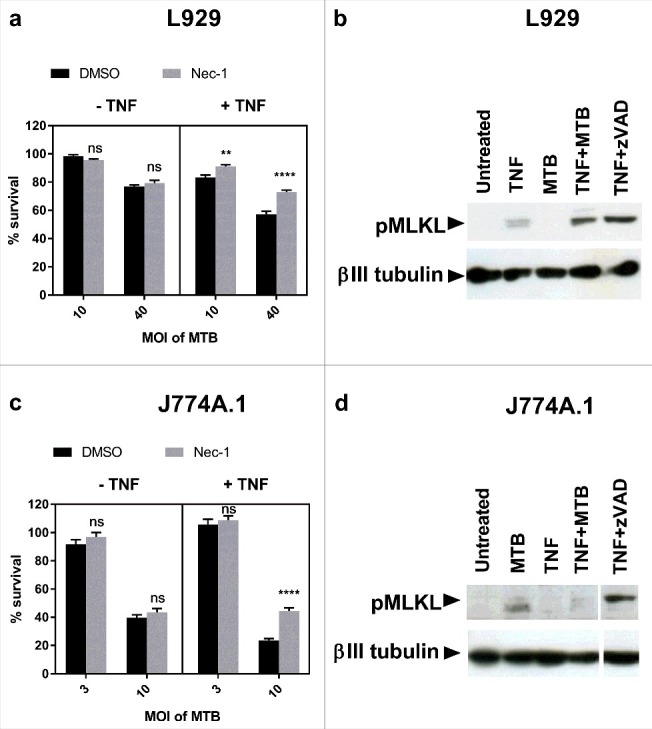
*M. tuberculosis* induces RIPK1-dependent cell death in the presence of excess TNFα in fibroblasts and macrophages, but only induces MLKL phosphorylation in fibroblasts. (a) L929 murine fibroblasts were infected with *M. tuberculosis* for 24 hours, then treated with DMSO or 30 µM Nec-1 in the presence or absence of 10ng/ml TNFα for 18 hours, before measuring cell survival using a crystal violet assay. Results are mean +/- SEM of n = 6 samples, and are expressed as a percentage of the uninfected control of each treatment. Statistics are two way ANOVA with Sidak post-test. Ns not significant; **p<0.01; ****p<0.0001. (b) Western blot of L929 fibroblasts infected with MOI 20 *M. tuberculosis* for 24 hours, then treated with TNFα for 24 hours, or with TNFα + 3 µM zVAD for 18 hours, developed with anti-pMLKL antibody and anti beta-III tubulin antibody. (c) J774A.1 murine macrophages were infected with *M. tuberculosis* for 3 hours, then treated with DMSO or 30 µM Nec-1 in the presence or absence of 25ng/ml TNFα for 48 hours. Results are mean +/- SEM of n = 10 samples, and are expressed as a percentage of the uninfected control of each treatment. Statistics are two way ANOVA with Sidak post-test. Ns not significant; ****p<0.0001. (d) Western blot of J774A.1 cells infected with MOI 10 *M. tuberculosis* for 3 hours and subsequently treated with 25ng/ml TNFα for 24 hours, or with TNF + 30 µM zVAD for 18 hours, developed with anti-pMLKL antibody and anti beta-III tubulin antibody. (a-d) All results are representative of at least 2 similar experiments.

Having demonstrated that L929 fibroblasts and J774A.1 macrophages are able to undergo necroptosis, we next investigated the presence of necroptosis in *M. tuberculosis* infection of these cell types. L929 fibroblasts and J774A.1 macrophages were infected with *M. tuberculosis* in the presence and absence of TNFα and the RIPK1 inhibitor Nec-1. Cell survival was determined using a crystal violet assay. As seen in Fig. 2a and Fig. 2c, *M. tuberculosis* induced cell death in both L929 fibroblasts and J774A.1 macrophages in a dose-dependent manner. In the absence of TNFα this cell death was not inhibited by Nec-1 and thus was not dependent on RIPK1. Addition of TNFα to the cultures following infection induced an additional proportion of cell death that was inhibited by Nec-1, demonstrating RIPK1-dependent cell death in both cell lines in the combined presence of TNFα and *M. tuberculosis* infection. We next investigated the ability of *M. tuberculosis* and TNFα to induce phosphorylation of MLKL at Ser345 (pMLKL). The Western blot in Fig. 2b shows *M. tuberculosis* and TNFα synergise to induce necroptosis via phosphorylation of Ser345 of MLKL in L929 fibroblast cells. However, despite the capability of J774A.1 to undergo necroptosis, and the occurrence of RIPK1-dependent cell death in *M. tuberculosis* and TNFα treated macrophages, these dying cells did not undergo phosphorylation of MLKL (Fig. 2d). Thus, we conclude that *M. tuberculosis* does not induce classical necroptosis in this macrophage cell type.

**Figure 3.**
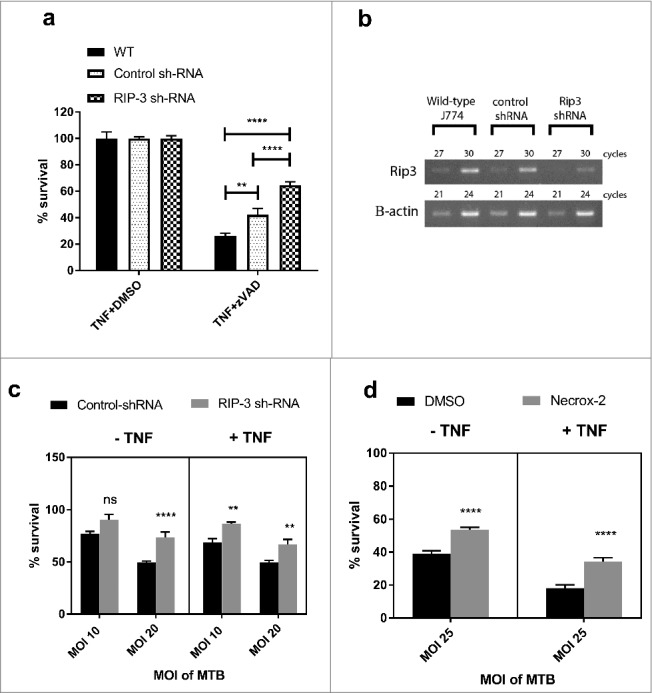
RIPK3 and mitochondrial ROS mediate M. tuberculosis-induced cell death both in the presence and absence of TNFα. (a) WT J774A.1 macrophages, RIP3K shRNA knockdown J774A.1 macrophages, and control shRNA J774A.1 macrophages were treated for 20 hours with DMSO control or 30 µM zVAD.fmk in the presence of 25ng/mL TNF, before measuring cell survival using a crystal violet assay. Results are mean +/- SEM of n = 10 samples and are expressed as a percentage of the TNFα+DMSO-treated control for each condition. Statistics are two way ANOVA with Sidak post-test. **p<0.01; ****p<0.0001. Results are representative of 3 independent experiments. (b) Knockdown of RIP3K mRNA was confirmed by RT-PCR, using murine RIP-3 and beta-actin primers (sc-61483-PR and sc-29192-PR, Santa Cruz). (c) RIP3K hRNA knockdown J774A.1 macrophages, and control shRNA J774A.1 macrophages were infected with *M. tuberculosis* for 3 hours, and subsequently incubated in the absence or presence of 25ng/ml TNF for 48 hours, before measuring cell survival using a crystal violet assay. Results are mean +/- SEM of n = 10 samples, and are expressed as a percentage of the uninfected control of each treatment. Statistics are two way ANOVA with Sidak post-test. *p>0.05; ***p<0.001; ****p<0.0001. Results are representative of 2 independent experiments (d) J774A.1 macrophages were infected with *M. tuberculosis* for 3 hours and incubated with Necrox-2 in the presence and absence of TNFα for 24 hours, before measuring cell survival using a crystal violet assay. Results are mean +/- SEM of n = 10 samples, and are expressed as a percentage of the uninfected control of each treatment. Statistics are one way ANOVA with Tukey's post-test. *p>0.05; ***p<0.001; ****p<0.0001.

We further sought to characterise the involvement of other effector molecules of the necroptotic pathway in macrophage cell death in response to *M. tuberculosis* infection in the presence and absence of TNFα. In the *M. marinum*/zebrafish model of tuberculosis, TNFα excess leads to RIPK1-RIPK3 dependent cell death mediated through phosphoglycerate mutase family member 5 (PGAM5) and mitochondrial reactive oxygen species (ROS) production.^43^ We therefore investigated the role of RIPK3 in J774A.1 macrophages by silencing the *RIPK3* gene using sh-RNA (Fig. 3a-b). As seen in Fig. 3c, RIPK3-deficient macrophages were protected from cell death induced by *M. tuberculosis*, however this effect was independent of TNFα signalling. Furthermore, addition of the mitochondrial ROS inhibitor Necrox-2 (Fig. 3d) was similarly able to rescue a proportion of cell death in *M. tuberculosis* infected macrophages, and this effect was similarly independent of TNFα signalling. Thus although pMLKL-driven necroptosis through TNFα-signalling does not occur in murine macrophages, pharmacologically tractable programmed necrosis driven by RIPK3 and mitochondrial ROS does occur in this cell type in response to *M. tuberculosis* infection.

**Figure 4.**
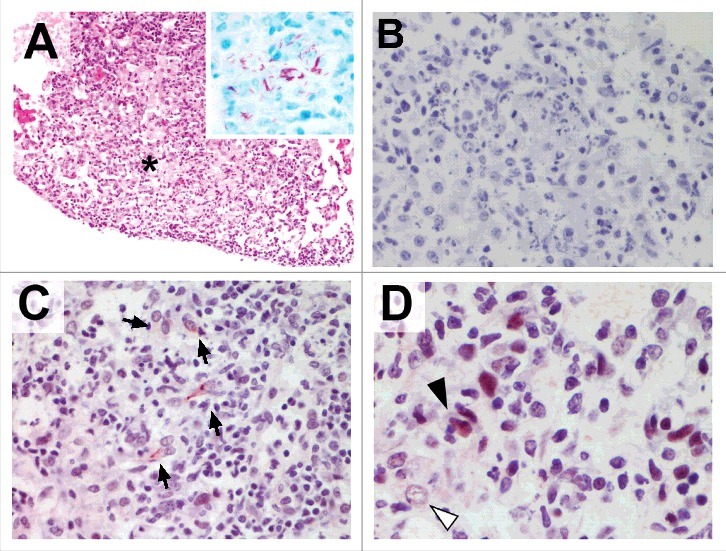
Murine tuberculosis granulomas contain non-macrophage cells undergoing pMLKL driven necroptosis. Mice were infected with *M. tuberculosis* by intranasal challenge and lung pathology analysed at 21d.p.i. (A) H&E stain. Granulomatous inflammation within the lung with abundant foamy macrophages and small areas of necrosis (asterisk). Original magnification: 100X. Inset shows Ziehl-Neelsen staining revealing numerous acid fast bodies (AFBs) present within the lesion. Original magnification: 400X. (B) Non-immune 1 ͦ antibody isotype control staining for immunohistochemistry. Original magnification: 400X. (C) pMLKL immunohistochemistry. Positive staining within the cytoplasm of elongated cells within the granulomatous inflammation and necrosis. Original magnification: 400X. (D) pMLKL immunohistochemistry. Positive staining is observed predominantly in cells with fusiform-nuclei (black arrow) adjacent to non-immunoreactive foamy macrophages (white arrow) and necrotic cell debris. Original magnification: 400X.

We finally sought to investigate the presence of pMLKL-driven necroptosis *in vivo* in mice infected with *M. tuberculosis*. Granulomatous TB lesions were immunostained for pMLKL. As seen in Fig. 4a, granulomatous regions were heavily consolidated with abundant acid fast bacilli (Fig. 4a inset), morphologically identifiable foamy macrophages and areas of necrosis. pMLKL immunohistochemistry of the *M. tuberculosis* infected lungs demonstrated necroptosis specifically in cells in granulomatous regions with necrotic debris (Fig. 4c-d). Both nuclear and cytoplasmic staining was observed in these cells, consistent with the scenario that upon activation, pMLKL translocates first to the nucleus and then to the cytoplasm.^33^ It is not possible in our study to definitively determine the identity of pMLKL-positive cells but it was notable that obvious foamy macrophages were pMLKL negative even in areas where proximal cells were pMLKL-positive (Fig. 4d, white arrow). Additionally, pMLKL-positivity tended to occur in cells with a fusiform nuclear and cell body morphology which is consistent with non-professional phagocytes such as fibroblasts of the lung stroma.^2^

## Discussion

There is compelling evidence that *M. tuberculosis* exerts a finely balanced control over the mode of death of its parasitized host cell.^19,50^ A number of studies show that following a period of apoptosis inhibition, infected macrophages undergo a form of death that resembles necrosis. This is supported by the experimental observations that low multiplicities of infection with virulent *M. tuberculosis* inhibit macrophage apoptosis,^51^ whereas high multiplicities of infection result in necrosis.^19,52,53^ The association of this phenomenon with *virulent* mycobacterial strains^19,20,23,54,55^ has led to a postulated scenario that necrosis is a virulence mechanism which enables bacterial escape to infect new phagocytes or into the extracellular milieu, concurrent with the generation of inflammation to drive the development of a necrotic granuloma which is essential for transmission. As is the case for many virulence features of *M. tuberculosis,* there appears to be a plethora of mechanisms that contribute to the regulation/inhibition of apoptosis. These may include ligation of TLR2 receptors by bacterial ligands such as lipoarabinomannan (LAM) and the 19KDa lipoprotein,^56,57^ the activities of bacterial NuoG^58^ and SecA2,^59^ and upregulation of host anti-apoptotic proteins Mcl-1^60^ and the Bcl-2 family member bfl-1/A1.^61^ However, there is a paucity of information regarding the mechanisms by which *M. tuberculosis* infected cells undergo necrosis, although individual molecular players are being discovered such as Rv2626c and PPE68; these genes contribute to induction of mitochondria-driven necrosis and enhance bacterial escape from the macrophage.^62^

There are likely a number of ways that *M. tuberculosis* causes cellular necrosis, but here we demonstrate for the first time that in a microenvironment of excess TNFα, *M. tuberculosis*-infected murine fibroblasts undergo necroptosis via RIP1K- and pMLKL-dependent mechanisms. Murine macrophages undergo RIPK1-dependent necrosis-like cell death, but do not undergo necroptosis because the scenario was not associated with phosphorylation of the MLKL death effector. We additionally demonstrate that necroptosis occurs *in vivo* in murine lung granulomas, where foamy macrophages lack pMLKL yet were spatially close to pMLKL-expressing cells including those with fusiform nuclei resembling fibroblasts.^2^

Necroptosis has been implicated as a mechanism of cell death in response to a range of micro-organisms including murine cytomegalovirus, Vaccinia virus, *Salmonella typhimurium*, and *Mycobacterium marinum*.^43,63^ However, direct comparison between these studies is complicated by varying definitions of “necroptosis”, as not all studies demonstrate definitive phosphorylation of MLKL. In the *M. marinum/*zebrafish model of tuberculosis, TNFα excess leads to RIPK1-RIPK3 dependent cell death, involving PGAM5 and mitochondrial ROS production.^43^ Although use of the MLKL inhibitor necrosulfonamide suggested a role for MLKL in programmed cell death in this scenario, definitive phosphorylation of MLKL was not demonstrated. Furthermore, recent phylogenetic analysis has shown that the members of the necroptotic signalling pathway are not well conserved through the animal kingdom; notably, MLKL is not present in zebrafish, suggesting that the *M. marinum*/zebrafish model does not fully replicate the programmed necrosis pathways of *M. tuberculosis*/macrophage infections when investigating pMLKL-driven necroptosis.^64^ Of further importance, not all cell lines are susceptible to necroptosis induced by TNFα and zVAD, which has been shown in some cell types to correlate with RIPK3 expression.^27^ In our hands, human primary macrophages, human U937 cells, murine J774A.1 cells and murine L929 cells were sensitive to TNFα+zVAD treatment, whereas THP-1 macrophages and RAW macrophages were not. Furthermore, the ability of J774A.1 macrophages and L929 fibroblasts to undergo pMLKL- dependent necroptosis was confirmed by Western blot, demonstrating their suitability for studying necroptosis. As necrotic pathways were the focus of this study, high MOIs (10-40) were used to infect macrophages and fibroblasts; cell death was observed to occur in a dose-dependent manner. Infection of macrophages with low MOI (MOI 3) was not able to stimulate RIPK1-dependent cell death in the presence of TNFα. It would be interesting to determine whether necroptosis occurs at low multiplicities of infection (when inhibition of apoptosis occurs) or whether it is a necrotic death phenotype that is exclusively dependent on a high multiplicity of infection.

During the preparation of this manuscript, Zhao *et. al.* published an elegant study demonstrating a key role of RIPK3 in inducing necrosis in *M. tuberculosis*-infected macrophages.^65^ They demonstrate that during infection of macrophages with virulent *M. tuberculosis*, a complex of RIPK1/RIPK3/pro-caspase-8 translocates to the mitochondria. Due to the presence of RIPK3 and Bcl-x_L_ at the mitochondrial membrane, pro-caspase-8 remains in its inactive zymogen form; BAK/BAX activation is not initiated and this results in an inhibition of intrinsic apoptosis. However, RIPK3 is able to stimulate ROS-dependent necrosis by enhancing the binding of hexokinase II to the voltage dependent anion channel (VDAC) on the mitochondrial membrane, and by triggering cyclophilin-D (CypD)-dependent formation of the MPT pore via interaction of VDAC and mitochondrial adenine nucleotide translocator (ANT). Both mechanisms are required for increased ROS formation and necrosis, and deficiency in RIPK3 resulted in enhanced survival of macrophages infected with *M. tuberculosis*. Interestingly, although MLKL-expression is increased in macrophages by *M. tuberculosis* infection, and MLKL-knockdown inhibited cell death in *M. tuberculosis*-infected macrophages, evidence of MLKL phosphorylation was not directly provided by this study. Our data complement and extend these findings. We demonstrate RIPK3 and mitochondrial ROS-dependent cell death occurs in macrophages both in the presence and absence of TNFα, but that de facto phosphorylation of MLKL does not occur in this cell type in response to *M. tuberculosis* infection, either *in vitro* or *in vivo* in murine tuberculosis granulomas. Furthermore, RIPK3-deficiency protected macrophages against *M. tuberculosis*-induced cell death. However, we demonstrate that pMLKL-driven necroptosis does occur in fibroblasts *in vitro* in the presence of TNFα, and that pMLKL-driven necroptosis can be detected *in vivo* in murine TB lung granulomas, predominantly in non-macrophage cell types resembling fibroblasts.

*M. tuberculosis* infection of fibroblasts presents a particularly intriguing conundrum. Fibroblasts are recruited to TB granulomas where they are involved in tissue remodelling, and *M. tuberculosis* is able to replicate in fibroblasts *in vitro*.^47,48^ *In situ* PCR has demonstrated *M. tuberculosis*-infected fibroblasts in latently infected individuals, suggesting that fibroblasts and other non-professional APCs could contain a reservoir of bacteria.^2^ However, infected fibroblasts are not seen in active *M. tuberculosis* infection. This has previously been attributed to the toxic effects of TNFα in active *M. tuberculosis* infection, where immune competent patients have high levels of circulating TNFα and pyresis.^47,48^ Our data demonstrate that the synergistic effects of *M. tuberculosis* infection and TNFα can cause necroptosis in fibroblasts, implicating necroptosis as the mechanism for enhanced toxicity of TNFα in *M. tuberculosis*-infected fibroblasts and potentially as the reason why *M. tuberculosis* infected fibroblasts are not frequently observed in active tuberculosis. Furthermore, as proliferation of fibroblasts and a fibrotic response has been shown to be critical to the encapsulation of the granuloma and control of tuberculosis infection, this raises the possibility that the induction of necroptosis in fibroblasts by *M. tuberculosis* and TNFα may provide an escape route for the pathogen from the encapsulated granuloma and therefore aid bacterial dissemination and transmission.^66^

Our data support a strategy to pharmacologically target programmed necrosis in active *M. tuberculosis* lesions.^25^ Active tuberculosis is characterised by necrotic lung damage, which is both detrimental to the host and aids release of viable bacteria and their transmission. Targeting programmed necrosis in macrophages or the necroptosis pathway in fibroblasts may protect these cells from the toxic effects of *M. tuberculosis* and TNFα, preventing necrotic tissue damage, inhibiting cavitation and augmenting participation in tissue remodelling.

## Materials and methods

### Bacterial culture

*M. tuberculosis* GC1237 and H37Rv was grown at 37°C in Middlebrook 7H9 broth containing 10% albumin/dextrose/catalase (ADC) plus 0.1% Tween-80 or on Middlebrook 7H11 medium containing 0.2% glycerol and 10% oleic acid/ADC (OADC) enrichment (Becton Dickinson). For macrophage infections, bacteria were grown to late log phase (an OD_600nm_ of 0.8-1.2) in 7H9 broth as described above, washed 1x in PBS-Tween-80 0.05% and once in PBS before resuspension in RPMI 1640 complete medium. OD_600nm_ was used to estimate bacterial numbers, with OD_600nm_ = 1 = 1 × 10^8^ cfu/ml.

### Macrophage and fibroblast cell culture

J774A.1 murine macrophage-like cells, L929 murine fibroblasts, THP-1 human monocytic cells and U937 human monocytic cells were cultured in RPMI 1640 complete medium containing 10% heat inactivated fetal calf serum (FCS) and 5 mM L-glutamine. RAW264.7 murine macrophage-like cells were grown in DMEM complete medium containing 10% FCS and 5 mM L-glutamine. J774A.1, L929 and RAW264.7 cells were seeded in 96-well plates and grown overnight at 37°C in 5% CO2 before mycobacterial infections and treatments to induce cell death. THP-1 and U937 monocytes were differentiated to macrophages using 25ng/ml PMA for 72 hours, then washed and rested for 24 hours before treatments to induce cell death.

Human PBMCs were isolated from component donation blood cones (NHS Blood and Transplant Service) by density centrifugation using Ficoll-Histopaque. The PBMC fraction was harvested and monocytes isolated with CD14 MicroBeads (Miltenyi Biotec). Monocytes were differentiated to macrophages in complete RPMI supplemented with 1% sodium pyruvate and 1% penicillin/streptomycin in 24 or 96 well plates with 20ng/mL of macrophage colony-stimulating factor (M-CSF; Miltenyi Biotec). Fresh medium with cytokines was added at day 3 and macrophages were used for further experiments after 6 days.

### Cell Survival Assays

Cells in 96 well plates were treated with TNFα (0-25ng/ml; Miltentyi Biotec), zVAD-fmk (30 µM, Promega), necrostatin (30 µM, Enzo) or Necrox-2 (30 µM Enzo) as indicated. For mycobacterial infections, cells were infected with GC1237 for the times indicated in figure legends, and the bacteria removed by gently washing once with warm PBS-1% FCS before replacing with complete medium containing the treatments as indicated. Plates were then washed 2x with PBS, fixed for 24 hours with 4% paraformaldehyde, washed with PBS and stained with crystal violet as previously described.^19^

### Confocal microscopy for cell morphology

J774A.1 cells were seeded in slide flasks, and the following day treated with TNF 25ng/ml, zVAD 30μM, Nec-1 30μM, 0.5mM H_2_O_2_, and 2.5μg/ml cyclohexamide. After 20h, cells were washed twice with PBS and fixed with 4% paraformaldehyde. Cells were washed and permeablised with 0.1% Triton-X100 for 3–5 minutes at room temperature, before staining with Alexa568-phalloidin (Molecular Probes) and DRAQ5 (Biostatus), mounting and viewing with a Zeiss LSM510META confocal microscope.

### Western blot

Following induction of cell death or mycobacterial infection, cells were lysed with RIPA buffer containing protease inhibitor cocktail, 2mM PMSF and 1mM sodium orthovanadate. *M. tuberculosis*-infected lysates were passed twice through 0.22 µm spin filters (Corning Costar Spin X) before processing under biosafety level 1 conditions. Protein concentration was measured using a BCA kit (Pierce) and 20 µg protein was subjected to reducing SDS-PAGE on 10% Bis-Tris gels (Novex, Invitrogen) and transferred to a 0.2 µm pore PVDF membrane. Membranes were blocked with 10% BSA in tris-buffered saline (TBS) for 1 hour at room temperature, rinsed with 0.1% Tween-20 (TBST), and incubated overnight at 4°C with primary antibodies specific for MLKL phosphorylated at Ser345 (EPR9515(2)) at a dilution of 1:2000, or anti beta III tubulin antibody (EP1331Y; Abcam) at a dilution of 1:10,000. Blots were rinsed thrice with TBST for 10 minutes, and incubated with the secondary reagent goat anti-rabbit IgG-peroxidase at a dilution of 1:6000 for 90 minutes at room temperature. Membranes were developed using Clarity ECL Western Blot Substrate (BioRad) and exposure to autoradiography film.

### RIPK3 shRNA knockdown cell lines

J774A.1 macrophages were transduced with lentiviral particles expressing shRNA of RIP3K (sc-61483-V) or control shRNA particles (sc-108080) according to the manufacturer's instructions (Santa Cruz). Transduced cells were selected with 1.25 µg/mL puromycin, and single cell colonies obtained by limiting dilution. Macrophages were washed and plated without puromycin selection for 24 hours prior to their use in assays for necroptosis or infection with *M. tuberculosis*. Knockdown of RIP3K mRNA was confirmed by RT-PCR, using murine RIP-3 and beta-actin primers (sc-61483-PR and sc-29192-PR, Santa Cruz).

### Mouse infections and immunohistochemistry

Animal experiments were performed in accordance with the Animals (Scientific Procedures) Act 1986. Female Balb/c mice were infected intranasally with approximately 2 × 10^2^ cfu of *M. tuberculosis* H37Rv (sample mean 2.13 × 10^2^), and sacrificed 21 days post infection. Excised lung tissue was fixed with 4% paraformaldehyde for 24 hours and embedded in paraffin wax. 4 µm tissue sections were dewaxed, rehydrated, endogenous peroxidase activity blocked by incubation with 3% H_2_O_2_ in methanol and epitopes demasked with proteinase K. Samples were incubated with 1:50 pMLKL antibody in TBS; sequential sections were run with rabbit IgG as an isotype control. Samples were incubated with biotinylated horse anti-rabbit IgG and antibody binding was amplified using avidin-biotin-peroxidase conjugate. Samples were developed with NovaRed substrate (Vector Laboratories), and counter stained with Mayer's haematoxylin. Acid fast bacilli in lung sections were visualised using Ziehl-Neelsen staining.

### Statistical analysis

One way ANOVA with Tukey's post-hoc tests, and two way ANOVA with Sidak post hoc test were performed using GraphPad Prism v6 software.
